# Theme Issue on E-Mental Health: A Growing Field in Internet Research

**DOI:** 10.2196/jmir.1713

**Published:** 2010-12-19

**Authors:** Heleen Riper, Gerhard Andersson, Helen Christensen, Pim Cuijpers, Alfred Lange, Gunther Eysenbach

**Affiliations:** ^7^University of TorontoDepartment of Health Policy, Management, and EvaluationToronto, ONCanada; ^6^University Health NetworkConsumer Health & Public Health Informatics LabToronto, ONCanada; ^5^University of AmsterdamDepartment of Clinical PsychologyAmsterdamNetherlands; ^4^The Australian National UniversityCentre for Mental Health ResearchCanberraAustralia; ^3^Linköping UniversityDepartment of Behavioural Sciences and LearningLinköpingSweden; ^2^GGZinGeestAmsterdamNetherlands; ^1^EMGO Institute of Health and Care ResearchVU University AmsterdamAmsterdamNetherlands

**Keywords:** editorial, e-health, e-mental health, randomized controlled trials, collborative care, feasibility study

## Abstract

This theme issue on e-mental health presents 16 articles from leading researchers working on systems and theories related to supporting and improving mental health conditions and mental health care using information and communication technologies. In this editorial, we present the background of this theme issue, and highlight the content of this issue.

## What is E-Mental Health?

We are pleased to introduce this JMIR Theme Issue on E-Mental Health. “E-mental health” can be understood as a generic term to describe the use of information and communication technology (ICT) –in particular the many technologies related to the Internet –when these technologies are used to support and improve mental health conditions and mental health care, including care for people with substance use and comorbid disorders. E-mental health encompasses the use of digital technologies and new media for the delivery of screening, health promotion, prevention, early intervention, treatment, or relapse prevention as well as for improvement of health care delivery (eg, electronic patient files), professional education (e-learning), and online research in the field of mental health. In a broader sense, all Internet interventions are, in one way or another, targeting the “mind” and human behavior. For the purpose of this theme issue, we focus on mental health conditions, while interventions that facilitate behavior change for general health problems (such as obesity) or systems that provide general emotional or social support for nonmental health conditions (eg diabetes) lie somewhat outside of the core scope of “e-mental health” (and this theme issue), although developers working in these areas can clearly learn from principles and findings presented in this issue.

Looking through the “e-mental health” articles published in JMIR in the past 3 years, it becomes evident that depression remains a major public health issue and a primary target for Internet interventions [1-9]. In addition, the question of whether Internet use itself has an impact on depression – a question fuelled by earlier studies on the apparent “Internet paradox” – has been studied [10]. Other conditions often targeted by e-mental health interventions are alcohol [11,12] and tobacco dependencies (a previous JMIR theme issue was devoted to Web-based tobacco interventions [13]) or conditions such as panic disorder [14] or pediatric encopresis [15]. Other recent e-mental health articles in JMIR have validated Web-based screening questionnaires for mental disorders [16] or explored the use of emerging technologies such as virtual reality [17] or mobile technologies [18].

## The First International E-Mental Health Summit

The current theme issue brings together a group of papers presented at the First International E-Mental Health Summit in Amsterdam in 2009 organized by the Trimbos Institute in collaboration with the International Society for Research on Internet Interventions (ISRII) [19], VU University Amsterdam, and the University of Amsterdam (UvA). The summit welcomed more than 500 people from over 40 countries.

The summit was preceded by the fourth ISRII meeting, which was opened by Professor Pim Cuijpers (VU University Amsterdam, Netherlands). He highlighted the need for international collaboration – a need illustrated by the growing numbers of ISRII participants. He symbolically transferred the ISRII chair to Professor Helen Christensen, who will lead ISRII up to its fifth meeting in Sydney, Australia, in April 2011.

Many young researchers presented their latest results during the ISRII meeting. Some of them moved beyond traditional cognitive-behavioral effectiveness studies. Björn Paxling (Linköping University, Sweden, and VU University Amsterdam, Netherlands), for example, ventured to compare Web-based cognitive-behavioral therapy (CBT) with Internet-delivered psychoanalytic therapy for general anxiety disorder (GAD). He found that the two interventions had comparable impacts in terms of clinical improvement. The ISRII 2009 Best Paper Award was won by Sylvia Gerhards (Maastricht University, Netherlands), who reported on one of the first economic evaluations of unguided online CBT for depression in primary care [20]. It favored online CBT alone in comparison with treatment as usual as well as treatment as usual supplemented by online CBT.

At the summit itself, 195 presentations were given by academics, health professionals, and policymakers, including some of the world’s most respected experts on eHealth intervention evaluation and dissemination research (see www.ementalhealthsummit.org). Presentations highlighted the effectiveness of Web-based treatment, new treatment developments, novel research methodologies, and the need for international collaboration. The summit was opened by Dr. Annemiek van Bolhuis, a deputy of the Dutch Minister of Health, who spoke of the importance of e-mental health as a response to growing demand and rising costs in mental health care. Dr. Jan Walburg, chair of the board of the Trimbos Institute, stressed the importance of mental well-being in the life cycle and the potential role of information and communication technologies in fostering it.

At the close of the summit, Professor Isaac Marks (University College London, London, United Kingdom) and Professor Alfred Lange (University of Amsterdam, Netherlands) were honored by the ISRII board as founding fathers of e-mental health in research and practice. It was also in Amsterdam that Professor Lange introduced in 1999 the first Web-based treatment program for people with posttraumatic stress disorders, known as Interapy.

This theme issue illuminates the evolving fields of eHealth and e-mental health, including topics such as the need for international collaboration, ethical considerations, cost-effectiveness, treatment attrition, and self-management.

**Figure 1:**
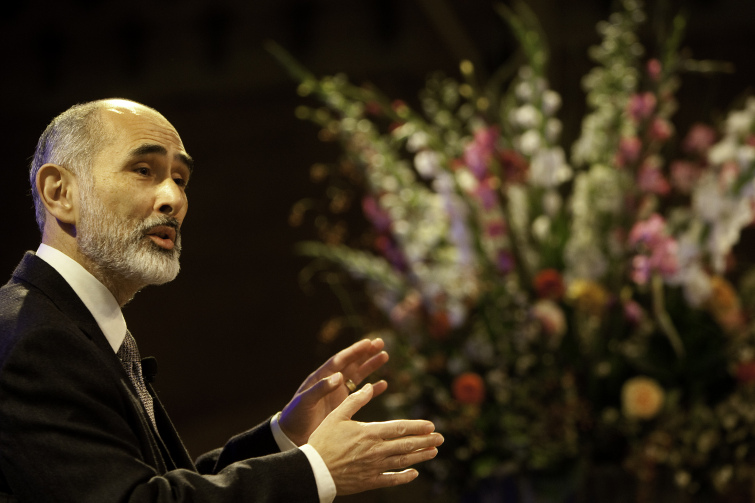
Professor Ricardo Muñoz at the E-Mental Health Summit

## Articles in This Theme Issue

### International Collaboration and Ethical Issues

Professor Ricardo Muñoz won the best presentation award, and we open this theme issue with his paper [21]. Echoing the enthusiasm of this international summit, he calls for smart international collaboration to disseminate Web-based interventions (eg, through portals like Beacon, www.beacon.anu.edu.au). Given the wide-scale, low-cost implementation potential of Internet interventions, the international dissemination of evidence-based interventions could help to reduce health disparities worldwide. Muñoz offers a framework for such an endeavor under the inspiring slogan, “Think globally, act locally, and share globally.”

In the study by Lange and Ruwaard [22], the authors demonstrate that sexually abused adolescents can be treated effectively in online treatment, but pretreatment withdrawal is frequent. These authors focus on several still unresolved ethical dilemmas in eHealth research. While adolescents are overwhelmingly present in the digital world, recruiting vulnerable adolescents to take part in outcome studies is hindered by a lack of perceived anonymity and the need to obtain permission and informed consent from their parents. Lange and Ruwaard also demonstrate that altering study designs may decrease the withdrawal rate. Full anonymity, however, is not a viable solution, either legally or professionally. The authors end their paper with a list of smart strategies to bolster youth participation in evaluation trials.

Another important ethical imperative, dealing with privacy and security, is discussed by Bennett and colleagues [23]. According to the authors, many interventions do not yet satisfy the necessary technical or other security requirements. One possible way to bring these security issues center stage is to apply an “ongoing risk assessment” design principle and to develop multidimensional security standards. The achievement of such aims could be greatly furthered by international collaboration.

### Cost-effectiveness

Cost-effectiveness trials are long overdue. In this issue, Warmerdam and colleagues [24] present evidence from one of the first such studies on Web-based, low-intensity guided self-help for depression. They assess the cost-utility and cost-effectiveness of problem-solving and cognitive-behavioral interventions for depression as compared with a wait-listed control group. Both interventions prove cost-effective, with no clear advantage for either the problem-solving or the cognitive-behavioral approach.

Online self-administered questionnaires for screening and for routine outcome assessment by patients themselves could also boost cost-effectiveness, but these questionnaires need to be validated when applying them in an online environment, as shown by Holländare and colleagues [25]. In a clinical sample, these authors compared the psychometric properties of online versions of Beck Depression Inventory (BDI-II) and the Montgomery-Åsberg Depression Rating Scale—Self-rated (MADRS-S) with the respective pen-and-paper versions. They conclude that both of these tests can be effectively delivered online, with the advantages of easy administration, dissemination, and data analysis. This, in turn, could both strengthen patient self-management and enhance clinicians’ knowledge, with a potential for improved clinical outcomes and cost savings.

White and colleagues [26] conducted a systematic review to assess the effectiveness of Web-based interventions for problem drinking by college students and adults. They found small to moderate effect sizes and conclude that Web-based interventions may be of particular interest to groups less likely to access traditional services, such as young people and women.

Of the contributors in this issue, two [27, 28] conducted randomized controlled trials of problem drinking among European young people, thereby widening the geographic scope of Web-based evaluation research of problem drinking beyond the United States. Contrasting with the review findings of White et al are those presented by Spijkerman and her coauthors concerning a younger group (ages 15 through 20) than those normally included in college trials [27]. Although the authors did not find a main effect on drinking outcomes associated with the Web-based intervention, it did appear to benefit males as compared with females in the short-term. Bewick and colleagues [28] evaluated the effectiveness of a personalized normative feedback intervention for alcohol use among university students at four institutions, finding positive results consistent with those obtained by White [26]. An interesting additional finding was the favorable influence on drinking outcomes in the assessment-only condition.

Parental skills were assessed in a pretest-posttest study by Van der Zanden and colleagues of a Web-based intervention aimed at parents with psychological problems [29]. These authors found improvement in parental competencies but no impact on child well-being. They conclude that the latter outcome may be attributable to the nonclinical baseline status of two thirds of the children, leaving little space for clinical improvement.

### Treatment Outcomes and Attrition

The high levels of study and treatment attrition (dropout) are hot and debated issues in eHealth and e-mental health intervention research, as exemplified by four papers in this issue. Applying theoretical considerations and empirical data, the authors build further on what Eysenbach has labeled the “law of attrition” [30].

Study dropout from eHealth interventions may not be random, and, if not adequately addressed, dropout may lead to biased estimations. Blankers and colleagues [31] provide an overview of imputation techniques for handling missing data—highly readable for nonexperts in statistics. They make use of data from a prospective cohort study of 124 participants in a self-help course for problem drinking. They conclude that multiple imputation techniques are best to avoid biased interpretations.

Nicholas and colleagues [32] studied predictors of treatment adherence using data from a randomized clinical trial of an online psycho-education program for people diagnosed with a bipolar disorder, supplemented by subsequent qualitative interviews with noncompleters about their reasons for nonadherence. The authors first contextualize online treatment attrition arguing that, although high, it does not differ significantly from that in face-to-face psychotherapy. Predictors of attrition identified by these authors, such as young age and male gender, as well as better insights into participants’ needs may aid in devising strategies to improve treatment adherence.

Mohr and colleagues [33] undertook a feasibility study in the community to investigate how adherence rates for online depression treatment, as well as the treatment outcome itself, could be improved by using multimodal functionalities. They used protocol-driven telephone coaching and Internet support based on persuasive technology. Their results, though tentative because of their single-arm design and small sample, revealed a low attrition rate in this online intervention, decreased depression scores, and high patient acceptability. In an interesting observation from a self-management point of view, they argue that the poorer moods associated with more intense use of the intervention may reflect patients’ titration of treatment in accordance with their own needs.

Meglič and colleagues [34] conducted a feasibility trial of primary-care depression treatment, investigating how collaborative care and online self-management support could improve medication adherence and lead to better clinical outcomes. As in the Mohr study [33], Meglič and colleagues found improved adherence rates, reduced depressive symptoms, and greater patient satisfaction. Notwithstanding these positive results, about one third of the sample reported drawbacks due to issues that included practicability. The results of both studies have prompted randomized controlled trials to investigate the robustness of these pilot results.

### Patient Self-management, Preferences, and Acceptability

The importance of self-management is addressed directly or indirectly in all papers in this issue. Nonetheless, empirical knowledge about patients’ preferences for and usage of Web-based interventions is still in its infancy. A study by Proudfoot and colleagues sheds light on the acceptability to patients of using mobile phones for the self-assessment and self-monitoring of clinical progress [35]. Applying a triangulation method, they conclude that those experiencing mental health problems such as depression were particularly interested in such an approach; those not interested mainly disliked using mobile phones or feared a lack of privacy.

Klein and colleagues assessed the preferences of 1214 people aged 16 or older who used information websites on alcohol and other drugs [36]. Easy search facilities, open access, and validated content were highly valued by the users. Differences in preference were associated mainly with age and educational level.

Schrank and colleagues conducted semistructured interviews to investigate Internet use among 26 people with schizophrenia, which may be the first such study conducted with mental health patients [37]. The respondents used the Internet to obtain information about their illness. Their Internet behavior resembled that of people without mental health disorders, though some were liable to specific problems such as stimulus overflow. Respondents believed the Internet had the potential to favorably change patients’ attitudes toward medication and their relationships with doctors.

The guest editors would like to thank all of the authors, who contributed their high-quality papers to this issue. We are also grateful to the reviewers for their helpful and pertinent comments on the submitted papers. Last but not least, we hope the readers will enjoy this theme issue of JMIR. It shows that Internet intervention research is clearly coming of age, though it still faces many challenges. And we hope to welcome you all to the fifth ISRII meeting in Sydney in 2011.

*Theme Issue Guest Editors*: Heleen Riper, Gerhard Andersson, Helen Christensen, Pim Cuijpers, Alfred Lange

*JMIR Editor-in-Chief and Publisher*: Gunther Eysenbach

## Abbreviations

BDI-II: Beck Depression Inventory
CBT: cognitive behavioral therapy
GAD: general anxiety disorder
ISRII: International Society for Research on Internet Interventions
ICT: information and communication technology
JMIR: Journal of Medical Internet Research
MADRS-S: Montgomery-Åsberg Depression Rating Scale—Self-rated
UvA: University of Amsterdam
